# Leukocyte Telomere Length and Cardiac Structure and Function: A Mendelian Randomization Study

**DOI:** 10.1161/JAHA.123.032708

**Published:** 2024-01-31

**Authors:** Ahmed M. Salih, Ilaria Boscolo Galazzo, Gloria Menegaz, André Altmann

**Affiliations:** ^1^ William Harvey Research Institute, NIHR Barts Biomedical Research Centre, Queen Mary University of London UK; ^2^ Department of Population Health Sciences University of Leicester UK; ^3^ Department of Computer Science University of Zakho Kurdistan of Iraq Iraq; ^4^ Department of Computer Science University of Verona Italy; ^5^ Centre for Medical Image Computing, Department of Medical Physics and Biomedical Engineering University College London UK

**Keywords:** cardiac IDPs, Mendelian randomization, telomere, Genetics, Cardiovascular Disease

## Abstract

**Background:**

Existing research demonstrates the association of shorter leukocyte telomere length with increased risk of age‐related health outcomes including cardiovascular diseases. However, the direct causality of these relationships has not been definitively established. Cardiovascular aging at an organ level may be captured using image‐derived phenotypes of cardiac anatomy and function.

**Methods and Results:**

In the current study, we use 2‐sample Mendelian randomization to assess the causal link between leukocyte telomere length and 54 cardiac magnetic resonance imaging measures representing structure and function across the 4 cardiac chambers. Genetically predicted shorter leukocyte telomere length was causally linked to smaller ventricular cavity sizes including left ventricular end‐systolic volume, left ventricular end‐diastolic volume, lower left ventricular mass, and pulmonary artery. The association with left ventricular mass (*β* =0.217, P_false discovery rate_=0.016) remained significant after multiple testing adjustment, whereas other associations were attenuated.

**Conclusions:**

Our findings support a causal role for shorter leukocyte telomere length and faster cardiac aging, with the most prominent relationship with left ventricular mass.

**Nonstandard Abbreviations and Acronyms**
IDP: image‐derived phenotype
IV: instrumental variable
LD: linkage disequilibrium
LTL: leukocyte telomere length
LVESV: left ventricular end‐systolic volume
LVM: left ventricular mass
MR: Mendelian randomization
UKB: United Kingdom Biobank

**Clinical Perspective**

**What Is New?**

- This is the first 2‐sample Mendelian randomization study assessing the potential causal role of leukocyte telomere length and a wide range of metrics representing cardiac function and structure.
- Genetically predicted shorter leukocyte telomere is causally linked to smaller ventricular cavity sizes, particularly left ventricular mass.

**What Are the Clinical Implications?**

- The results of our study add a new risk factor to the vascular risk factors that should be considered to assess cardiac function and structure.

Global population aging has increased the burden of chronic noncommunicable diseases of older age, among which cardiovascular diseases (CVDs) are the most prominent.^1^ The risk of age‐related CVD is not uniform across the population. Indeed, biological cardiovascular aging is influenced by a wide range of environmental and lifestyle exposures throughout life.^2^ Furthermore, the susceptibility to CVDs varies even among individuals with similar exposure profiles, suggesting the crucial role of inherent genetic determinants of biological aging.

Telomeres are distinct regions of repetitive nucleotide sequences and protein complexes at the end of chromosomes.^3^ Their function is to protect against nucleotide degradation, unnecessary recombination, repair, and interchromosomal fusion.^4^, ^5^ Telomeres become progressively shorter with each cell division. Shorter leukocyte telomere length (LTL) has been linked to increased risk of death and age‐related disease, including a range of CVDs.^6^ For instance, longer LTL is causally associated with decreased risk of myocardial infarction, coronary atherosclerosis, and ischemic heart disease.^7^ There is wide variation of LTL across the population with evidence of high and consistent heritability.^8^, ^9^ As such, LTL has been proposed as an indicator of biological aging providing information beyond chronological age and environmental exposures. However, LTL can also be modified by diseases, such as diabetes and atherosclerosis, which may confound its relationships with incident CVD and death.^6^ That is, the causality of the relationships between LTL and CVD is not adequately demonstrated in existing literature.^10^

Cardiac magnetic resonance (CMR) became an essential tool for the characterization of CVDs. The cardiovascular phenotype alters in a predictable and established manner with increasing age, which may be accurately captured using CMR.^11^ Thus, CMR phenotypes provide reliable continuous measures of cardiovascular aging. The recent availability of genome‐wide association study (GWAS) data^12^, ^13^, ^14^, ^15^ for a rich array of CMR phenotypes provides opportunity to evaluating causal associations of LTL with cardiovascular aging.

A statistical genetics technique that can help establish causality from GWAS results is Mendelian randomization (MR). A recent study used MR to examine the association between LTL and 7 CMR metrics.^16^ The findings indicate that shorter LTL was associated with reduced left ventricular (LV) mass (LVM), reduced LV stroke volume, reduced global ventricular volume, and reduced overall ventricular size. However, only the reduction in LVM would survive multiple testing correction across the 7 explored traits. In particular, the study implemented a 1‐sample MR, where the exposure (LTL) and outcome (CMR metrics) are obtained from the same cohort, which enabled the authors to leverage a large data set comprising N=446 367 individuals to identify instrumental genetic instruments of LTL (exposure) for the MR analysis of whom N=40 459 underwent CMR (outcome). However, such analyses are known to be more prone to bias compared with 2‐sample MR.^17^ For instance, weak instruments bias the result toward the observed association between exposure and outcome,^17^ whereas winners curse may lead 1‐sample MR to underestimate the true causal effect.^18^ Therefore, a 2‐sample MR, where there is no overlap between the samples of the exposure and the outcome and which avoids such biases, can be used to substantiate these findings. Finally, Aung et al^16^ focused on the most common CMR metrics. However, there are further metrics, such as ejection fraction, which are of broad interest and can be explored using available GWAS summary statistics in a 2‐sample MR framework.

In this study we used 2‐sample MR to examine the causal association of LTL with 54 CMR phenotypes, leveraging publicly available GWAS summary statistics of LTL and cardiac phenotypes. This work provides novel insights into the causal associations of LTL with cardiovascular aging and its potential as a marker of biological cardiovascular age, independent of age and environmental exposures.

## Methods

The authors declare that all supporting data are available within the article and references.

### Study Design

The GWASs summary statistics that support this analysis are previously published and downloadable results from different projects/cohorts including UK Biobank (UKB) and European Network for Genetic and Genomic Epidemiology. Ethical approval and participants formal consent were collected and approved in each study from the respective institutions.

### Cardiac IDPs (Outcome) GWAS

#### Right Atrium

The GWAS summary statistics of the right atrium^12^ that are publicly available were considered in the current analysis. Right heart structure and function GWAS was conducted for 40 000 participants from UKB. Participants were excluded if they had history with specific heart diseases. GWAS analysis was conducted for 34 CMR measures including right ventricular volume at end diastolic and end systolic, ejection fraction, and stroke volume. In addition, Pirruccello et al^12^ conducted GWAS for the pulmonary system, including pulmonary root diameter, pulmonary artery, and diameter of pulmonary artery. Similar measures were also used but indexed for body surface area. Although their analysis was mainly focused on the right atrium, they also conducted GWAS for LV and right ventricular metrics. The full list of all 34 CMR measures reported and the GWAS results can be found in.^12^

#### Right Ventricular

To represent right ventricular phenotypes, we used publicly available GWAS summary statistics of the following 4 right ventricular measures: end systolic volume, stroke volume, ejection fraction, and end diastolic volume.^13^ In summary, the results are based on 29 506 participants from UKB free from preexisting myocardial infarction or heart failure.

#### Left Atrium

GWAS was performed using 5 functional and volumetric left atrial (LA) variables from UKB for 35 658 participants. The variables included LA minimum volumes, indexed LA maximum, LA active emptying fraction, LA passive emptying fraction, and LA total emptying fraction.^14^

#### Left Ventricular

We used the GWAS summary statistics for the following 6 LV measures: end‐diastolic volume, LVM, end‐systolic volume (LVESV), ejection fraction, mass to end‐diastolic volume ratio, and stroke volume.^15^ However, as the summary statistics of LV stroke volume were not available, this measure was excluded from further analyses. The study was conducted on 16 923 European participants from the UKB.

#### Aortic Distensibility

GWAS was conducted to assess the association of the genetic bases and 6 aortic dimension and distensibility phenotypes. These included ascending aortic distensibility, ascending aortic minimum area, ascending aortic maximum area, descending aortic distensibility, descending aortic minimum area, and descending aortic maximum area.^19^ The analysis was conducted on 32 590 White participants from the UKB.

#### Arterial Stiffness Index

Arterial stiffness index GWAS was conducted on 127 121 European‐ancestry individuals from the UKB.^20^ Such analysis has been facilitated by its ease of acquisition and its role in a wide range of CVDs. The analysis resulted in a set of variants in 4 loci that are significantly associated with arterial stiffness index.

### LTL (Exposure) GWAS

We used 33 single nucleotide polymorphisms (SNPs) that have been shown to be significantly associated with LTL in previous studies.^21^, ^22^, ^23^ The first 20 SNPs were from a recent study that was conducted on 78 592 European individuals, under the European Network for Genetic and Genomic Epidemiology (ENGAGE) prject. The remaining 13 SNPs were used before in^24^ to conduct MR analysis with a wide range of aging‐related outcomes. The focus on these studies, which did not use UKB data, for the selection of the instrumental variables (IVs) allows us to conduct a 2‐sample MR study using CMR GWAS summary statistics that were obtained using UKB participants. Accordingly, there was no overlap between the participants included in the exposure (LTL) and the outcome (CMR image‐derived phenotypes [IDPs]) GWASs. The process of selecting the IVs, linkage disequilibrium (LD) clumping, and performing MR analysis are motivated by the steps mentioned in.^25^

The process of choosing proxies for SNPs that are not available for the GWAS on CMR measures and ensure that they are not in LD was performed as described previously.^26^ Briefly, 10 SNPs were excluded as they were in high LD (R^2^>0.02) with other SNPs. Ensembl^27^ was used to calculate LD using GBR (British in England and Scotland) samples from phase 3 (version 5) of the 1000 Genomes Project. The final list of the 23 SNPs is shown in Table 1.

**Table 1:** SNPs Used in the MR Analysis

SNPs	Chr	Pos	Gene	EA	OA	EAF	Beta	SE	*P* value	Source
**rs2695242** ^*^	1	226 406 337	*PARP1*	G	T	0.83	−0.039	0.006	9.31E‐11	[21]
rs11125529	2	54 248 729	*ACYP2*	A	C	0.16	0.065	0.012	4.48E‐08	[22]
rs6772228	3	58 390 292	*PXK*	T	A	0.76	0.041	0.014	3.91E‐10	[28]
rs55749605	3	101 513 249	*SENP7*	A	C	0.58	−0.037	0.007	2.45E‐08	[21]
**rs7643115** ^*^	3	169 794 453	*TERC*	A	G	0.243	−0.085	0.0057	6.42E‐51	[21]
rs13137667	4	70 908 630	*MOB1B*	C	T	0.959	0.076	0.013	2.37E‐08	[21]
**rs7675998** ^*^	4	163 086 668	*NAF1*	G	A	0.8	0.048	0.012	4.35E‐16	[28]
rs7705526	5	1 285 859	*TERT*	A	C	0.328	0.082	0.005	4.82E‐45	[21]
rs34991172	6	25 480 100	*CARMIL1*	G	T	0.068	−0.060	0.010	6.03E‐09	[21]
**rs805297** ^*^	6	31 654 829	*PRRC2A*	A	C	0.313	0.034	0.0055	3.41E‐10	[21]
rs59294613	7	124 914 213	*POT1*	A	C	0.293	−0.040	0.005	1.12E‐13	[21]
rs9419958	10	103 916 188	*STN1 (OBFC1)*	C	T	0.862	−0.063	0.007	4.77E‐19	[21]
rs228595	11	108 234 866	*ATM*	A	G	0.417	−0.028	0.005	1.39E‐08	[21]
**rs76891117** ^*^	14	72 933 129	*DCAF4*	G	A	0.1	0.047	0.0084	1.64E‐08	[21]
rs3785074	16	69 373 083	*TERF2*	G	A	0.263	0.035	0.005	4.5E‐10	[21]
rs62053580	16	74 646 176	*RFWD3*	G	A	0.169	−0.038	0.007	3.96E‐08	[21]
rs7194734	16	82 166 375	*MPHOSPH6*	T	C	0.782	−0.036	0.006	6.72E‐10	[21]
rs3027234	17	8 232 774	*CTC1*	C	T	0.83	0.103	0.012	2E‐08	[23]
rs8105767	19	22 032 639	*ZNF208*	G	A	0.289	0.039	0.005	5.21E‐13	[21]
rs6028466	20	39 500 359	*DHX35*	A	G	0.17	0.058	0.013	2.57E‐08	[21, 23]
**rs71325459** ^*^	20	63 636 988	*RTEL1*	T	C	0.015	−0.139	0.022	7.04E‐10	[21]
rs75691080	20	63 638 397	*STMN3*	T	C	0.091	−0.067	0.008	5.75E‐14	[21]
rs73624724	20	63 805 045	*ZBTB46*	C	T	0.129	0.050	0.007	6.08E‐12	[21]

*Indicates proxies as the original SNPs were not available for cardiac image‐derived phenotype genome‐wide arassociationea studies. Gene: the closet candidate genes. The reported position is from GRCh38 genome build. SNPs, ID of the SNP. Beta indicates beta value of the SNP in GWAS; Chr, chromosome; EA, effect allele; EAF, effect allele frequency; MR, Mendelian randomization; OA, other allele; Pos, position of the SNP in the genome; and SNP, single nucleotide polymorphism.

### Statistical Analysis

The statistical analysis was implemented using TwoSampleMR package^29^ in R adopting inverse‐variance weighted method as the primary analysis. Weighted mode and weighted median methods were also performed as complementary MR analyses. The inverse‐variance weighted method assumes no pleiotropy whereas weighted mode and weighted median allow for genetic pleiotropy.^25^ In addition, weighted median presents consistent estimates when at least 50% of the selected variants are valid IVs.^30^ Moreover, weighted mode assumes that the true causal association is coming from common causal effect even if the majority of IVs are invalid.^31^ MR‐Egger regression^32^ and weighted median function^30^ were used to detect heterogeneity and directional pleiotropy of the genetic instruments. To test for horizontal pleiotropy test, leave‐one‐SNP‐out analyses, MR‐Egger intercept test, and the modified Cochran Q statistic methods were applied. Moreover, MR–Pleiotropy Residual Sum and Outlier^33^ was used to detect and correct pleiotropy. In addition, in order to further address the issue of potential pleiotropy, we conducted a sensitivity analysis after excluding any SNPs with evidence for a genome‐wide association with another trait. To this end we screened all IVs against the GWAS Catalog (https://www.ebi.ac.uk/gwas/; date accessed November 17, 2023) summary statistics. SNPs with *P* values below 5×10^−8^ for traits other than telomere length were considered for exclusion in a sensitivity analysis. False discovery rate^34^ correction was finally applied to the inverse‐variance weighted results to adjust for multiple tests across the 54 cardiac GWAS studies independently at *α*=0.05.

### Sensitivity Analysis

After our initial screen, we conduct a multivariable 2 sample MR on significant outcomes to ensure the effects are not driven by body mass index or smoking. To further assess the association between LTL and LVM, we ran multivariable 2‐sample MR to adjust the association for body mass index and smoking. Body mass index GWAS summary statistics were extracted from,^35^ which did not include UKB participants. In terms of smoking, we used summary statistics from,^36^ using cigarettes per day, without UKB participants and downloaded from (https://conservancy.umn.edu/handle/11299/201564). Thereafter, we run the analysis using the TwoSampleMR package^29^ and followed the instructions mentioned in (https://mrcieu.github.io/TwoSampleMR/articles/perform_mr.html) using the function mv_multiple().

## Results

In total 54 MR tests were conducted to examine the causal association of LTL and CMR phenotypes. All the IVs used from LTL GWAS were also available for cardiac IDPs, with the exception of 2 SNPs (rs13137667 and rs71325459) from the LV GWAS.^15^ We could not select suitable proxies for these 2 SNPs, because all variants in the region showed low LD (LD <0.01). Consequently, these 2 SNPs were excluded when MR was conducted between LTL and LV IDPs. Five measures showed nominally significant association with LTL in both the main and complementary analyses (Table 2). Specifically, we found significant causal association of shorter LTL with smaller LV cavity volumes in end‐diastole and end‐systole (lower LV end‐diastolic volume and LVESV) and lower LVM. Shorter LTL was also significantly associated with smaller short axis pulmonary artery size (in both indexed and nonindexed versions of this metric). There was no evidence of significant horizontal pleiotropy in these 5 measures, as per MR–Pleiotropy Residual Sum and Outlier (*P* value of global test was >0.05). In addition, the MR‐Egger intercept *P* value was not significant indicating no directional pleiotropy. The association with LVESV was not significant in one of the complementary analyses (weighted median), whereas it was significant in other analyses. The associations with short axis pulmonary artery size (in both indexed and nonindexed versions of this metric), LV end‐diastolic volume and LVESV were attenuated to statistically nonsignificant after multiple testing adjustment (*P*_false discovery rate_>0.05). The association with LVM was the most robust, remaining statistically significant after multiple testing adjustment (*P*_false discovery rate_=0.016). The remaining CMR measures were not significantly associated with LTL (Table S1). The results showed that shortening LTL over time causes reduction particularly in LVM and suggestively in 4 other measures. The Figure shows that LTL shortening causes reduction of all 5 cardiac IDPs over time.

**Table 2:** Results of the Mendelian Randomization for the CMR Measures Significantly Associated With LTL

Method	LVEDV	LVESV	LVM	LVM (SE)	sa_cm_pa	sa_cm_pa_i
IVW beta	0.138	0.118	0.217	0.24	0.085	0.103
IVW SE	0.054	0.054	0.061	0.067	0.049	0.007
IVW *P* value	0.011	0.029	0.0003	0.0002	0.049	0.007
IVW false discovery rate *P* value	0.198	0.392	0.016		0.486	0.189
MR‐Egger *P* value	0.011	0.027	0.048	0.01	0.012	0.006
Weighted median *P* value	0.003	0.141	0.005	0.004	0.007	0.005
Weighted mode *P* value	0.025	0.031	0.029	0.006	0.013	0.014
Pleiotropy tests						
Egger intercept *P* value	0.05	0.10	0.40	0.15	0.50	0.05
MR‐PRESSO (global test) *P* value	0.44	0.61	0.27	0.30	0.06	0.22
Source	[15]	[15]	[15]	[15]	[12]	[12]
Sample size	16 923	16 923	16 923	16 923	40 000	40 000

The reported *P* values of MR‐PRESSO are from the global test. i indicates indexed to body surface area; IVW, inverse‐variance weighted; LTL, leukocyte telomere length; LVEDV, left ventricular end‐diastolic volume; LVESV, left ventricular end‐systolic volume; LVM (SE), the association with left ventricular mass after removing the 3 instrumental variables; MR, Mendelian randomization; MR‐PRESSO, MR–Pleiotropy Residual Sum and Outlier; pa, pulmonary artery; and sa, short axis.

**Figure 1:**
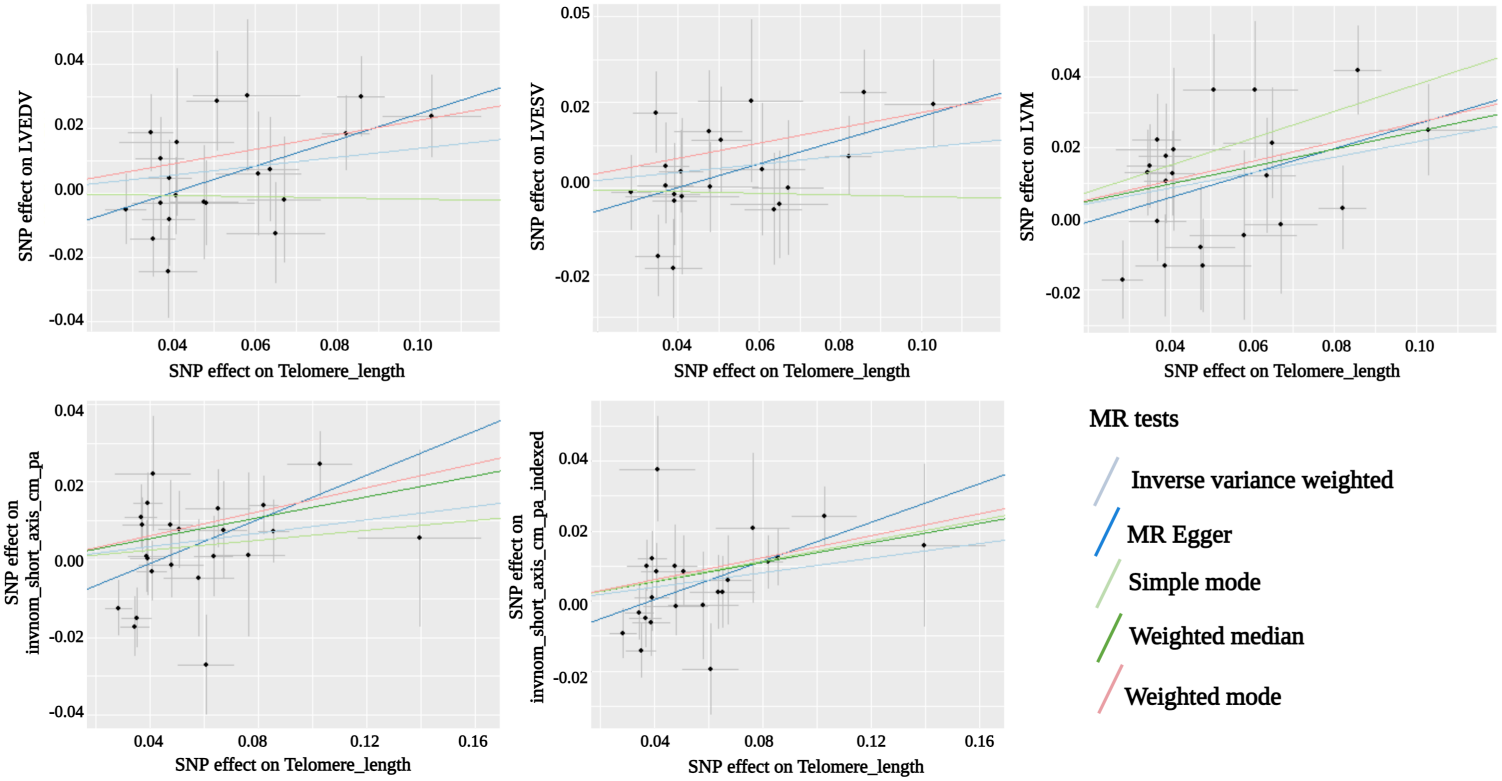
Association of LTL and the 5 cardiac IDPs in the primary (IVW) and in the complementary analyses. IDPs indicates image‐derived phenotypes; IVW, inverse‐variance weighting; LTL, leukocyte telomere length; LVEDV, left ventricular end‐diastolic volume; LVESV, left ventricular end‐systolic volume; LVM, left ventricular mass; MR, Mendelian randomization; and SNP, single nucleotide polymorphism.

For LVM we considered 2 sensitivity analysis. First, we repeated the analysis after excluding SNPs with a pleiotropic effect. The results from GWAS Catalog screening identified 3 IVs that are significantly associated with a wide range of traits, which might bias the MR analysis. The IVs are rs7705526 (with many traits including lung cancer and myeloid white cell count), rs76891117 (with systolic blood pressure), and rs7194734 (with body height; additional details in Table S2). Those 3 IVs were removed and the analysis was run again for LVM. The association with LVM remained significant after removing the 3 IVs (Table 2). In fact, the effect size was stronger than with the original set of IVs. Second, we conducted the multivariable 2‐sample MR between LTL and LVM adjusted for body mass index and smoking. The analysis also indicated a significant association (*β*=0.2001, *P* value=0.0184).

## Discussion

We present an extensive evaluation of the causal relationships between LTL and 54 cardiac IDPs using 2‐sample MR analysis. We demonstrate causal association of shorter LTL with smaller LV cavity volumes (smaller LV end‐diastolic volume and LVESV) and lower LVM. The most robust association was between LTL and LVM, with the association remaining significant after multiple testing correction of the primary analysis. The pattern of associations reflects associations of LTL with a pattern of cardiovascular phenotypic alterations in keeping with greater cardiac aging. These phenotypic alterations have been shown to confer significant cardiovascular risk, particularly when present at younger ages. Thus, our work supports a causal role for LTL in driving poorer cardiovascular health and sheds light into potential mechanisms causing differential susceptibility to CVD across the population. The pattern of phenotypic alterations observed in association with shorter LTL is consistent with age‐related cardiovascular changes reported in population imaging cohorts.^37^ There are distinct age‐related morphological alterations of the heart, which are reflected in cardiovascular imaging phenotypes and widely reported in the literature.^11^ Increasing age is associated with cardiomyocyte attrition,^38^ which is detected as lower LVM on CMR. There is also substantial decline in LV volumes with increasing age, which is disproportionately greater than the LVM decline, therefore resulting in a concentric pattern of LV remodeling in older ages.^37^ With regard to functional metrics, the pattern is more complex; poorer strain metrics are generally noted, although their measurement on CMR can be challenging. LV stroke volume is reduced with increasing age but accompanied by minimal or no change in LV ejection fraction. Increased vascular stiffness with older age is well described in the literature^39^ and linked to the described ventricular remodeling through patterns of ventricular‐arterial coupling.^40^ The described age‐related cardiovascular alterations have been linked to increased risk of cardiovascular events. Thus, the observed associations with shorter LTL in our study are well matched to existing knowledge on cardiac age‐related remodeling. It is worth mentioning that the rate of telomeres length shortening over time is different across cell types.^9^ Telomeres in proliferative cells such as leukocytes experience higher shortening rate compared with nonproliferative cells such as muscle cells.^41^ Accordingly, the rate of telomere length shortening in muscular tissue such as cardiac would be slower than in the other tissues.^42^

Although our analysis indicates a causal relationship between shorter LTL and age‐related LV remodeling, it is not clear whether this represents a direct effect of LTL or an indirect effect mediated through greater propensity to risk factors such as diabetes. Indeed, previous work has been linked shorter LTL to poorer glycemic control,^43^ higher blood pressure,^44^, ^45^, ^46^ and total cholesterol.^47^ It is likely that LTL acts through multiple direct and indirect pathways to accelerate cardiac aging and increase propensity to disease. Further studies elucidating these precise mechanisms are essential to advance knowledge in this area. The associations with right ventricular and atrial metrics were not significant in our analysis. This may reflect greater technical challenges in deriving these phenotypes and thus greater noise limiting detection of SNPs in GWAS for these metrics. For instance, the right ventricle has an irregular anatomy, and its segmentation is far more challenging than the LV with greater interoperator variation.^48^ It is also possible that the LV reflects more readily age‐related alterations than the other chambers. The associations of CMR metrics with aging are not extensively studied due to limited availability of large population cohorts. As the performance automated segmentation tools improve and with greater availability of population data sets our understanding of the genetic architecture of the heart is expected to improve with greater opportunity to understand its alterations with age.

The IVs (SNPs) used in our analysis belong to wide range of genes that regulate LTL and are also linked to the development of cardiac diseases and vascular risk factors. The rs11125529, rs7675998, rs8105767, and rs7194734 SNPs belong to the *ACYP2, NAF1, ZNF208*, and *MPHOSPH6* genes, respectively, which were found to be associated with increasing risk of developing coronary heart disease.^22^, ^49^, ^50^ In addition, rs6772228 belongs to the *PXK* gene, which is associated with total cholesterol levels.^51^ Finally, the *RTEL1* gene plays a protective role against coronary heart disease.^52^

Importantly, our study confirms the previously reported causal association between LTL and LVM^16^ using 2‐sample MR, which is less susceptible to bias compared with 1‐sample MR. Similarly to Aung et al,^16^ who reported a nominally significant effect between LTL and LV stroke volume, there were also significant associations between LTL and LV end‐diastolic and end‐systolic volume, but these did not survive multiple testing correction. Interestingly, our results were achieved using only 23 SNPs as IVs, which were previously reported to be significantly associated with LTL, as compared with the 130 SNPs by Aung et al^16^ based on a UKB GWAS analysis. Of note, the effect sizes in our study were larger than the ones previously reported using 1‐sample MR. For example, the effect size of LVM in our study is (*β*=0.21) per 1‐SD increase in genetically determined telomere length, whereas Aung et al^16^ report (*β*=0.13). Accordingly, the results of the current study provide additional robust evidence for a causal association between LTL and CMR metrics.

A constant concern in MR analyses is bias due to horizontal pleiotropy. We attempted to address this constraint in two ways. First, we used statistical methods that attempt to detect pleiotropy and adjust for pleiotropy (MR–Pleiotropy Residual Sum and Outlier). None of our nominally significant associations showed evidence of pleiotropy using this method. Second, we manually screened the IVs against a large database of published GWAS results (GWAS Catalog). Instruments that were found to be strongly related to other traits were removed as IVs in a sensitivity analysis. Across both these approaches our main result of LTL influencing LVM remained significant. However, despite these efforts we cannot fully exclude the risk of a bias in our MR results due to pleiotropy.

## Conclusions

To the best of our knowledge, this is the first study that attempts to assess the casual link between LTL and a wide range of cardiac IDPs. Our results indicate a significant and causal role of LTL on the LV structure. A previous study^2^ demonstrated the role of wide range of daily lifestyle and exposures in cardiac aging. Our study identified a new significant player in cardiac aging, which might help to better understand the aging‐driven factors.

## Sources of Funding

Ahmed M. Salih is supported by a British Heart Foundation project grant (PG/21/10619). Ilaria Boscolo Galazzo and Gloria Menegaz acknowledge support from Fondazione CariVerona (Bando Ricerca Scientifica di Eccellenza 2018, EDIPO project, num. 2018.0855.2019) and MIUR D.M. 737/2021: AI4Health: empowering neurosciences with eXplainable AI methods.

## Disclosures

None.

## Supporting information

Tables S1–S2

## References

[1] Roth GA , Mensah GA , Johnson CO , Addolorato G , Ammirati E , Baddour LM , Barengo NC , Beaton AZ , Benjamin EJ , Benziger CP , et al. Global burden of cardiovascular diseases and risk factors, 1990–2019: update from the GBD 2019 study. J Am Coll Cardiol. 2020;76:2982–3021. doi: 10.1016/j.jacc.2020.11.010 33309175 PMC7755038

[2] Raisi‐Estabragh Z , Salih A , Gkontra P , Atehortúa A , Radeva P , Boscolo Galazzo I , Menegaz G , Harvey NC , Lekadir K , Petersen SE . Estimation of biological heart age using cardiovascular magnetic resonance radiomics. Sci Rep. 2022;12:12805. doi: 10.1038/s41598-022-16639-9 35896705 PMC9329281

[3] Revy P , Ca K , Bertuch AA . Genetics of human telomere biology disorders. Nat Rev Genet. 2022;24:1–23. doi: 10.1038/s41576-022-00527-z 36151328

[4] Blasco MA . Telomeres and human disease: ageing, cancer and beyond. Nat Rev Genet. 2005;6:611–622. doi: 10.1038/nrg1656 16136653

[5] Blasco MA . Telomere length, stem cells and aging. Nat Chem Biol. 2007;3:640–649. doi: 10.1038/nchembio.2007.38 17876321

[6] Serrano AL , Andreós V . Telomeres and cardiovascular disease: does size matter? Circ Res. 2004;94:575–584. doi: 10.1161/01.RES.0000122141.18795.9C 15031270

[7] Deng Y , Li Q , Zhou F , Li G , Liu J , Lv J , Li L , Chang D . Telomere length and the risk of cardiovascular diseases: a mendelian randomization study. Front Cardiovasc Med. 2022;9:1012615. doi: 10.3389/fcvm.2022.1012615 36352846 PMC9637552

[8] Broer L , Codd V , Nyholt DR , Deelen J , Mangino M , Willemsen G , Albrecht E , Amin N , Ma B , de Geus EJC , et al. Meta‐analysis of telomere length in 19 713 subjects reveals high heritability, stronger maternal inheritance and a paternal age effect. Eur J Hum Genet. 2013;21:1163–1168. doi: 10.1038/ejhg.2012.303 23321625 PMC3778341

[9] Demanelis K , Jasmine F , Chen LS , Chernoff M , Tong L , Delgado D , Zhang C , Shinkle J , Sabarinathan M , Lin H , et al. Determinants of telomere length across human tissues. Science. 2020;369:eaaz6876. doi: 10.1126/science.aaz6876 32913074 PMC8108546

[10] Mather KA , Jorm AF , Parslow RA , Christensen H . Is telomere length a biomarker of aging? A review. J Gerontol A Biol Sci Med Sci. 2011;66:202–213. doi: 10.1093/gerona/glq180 21030466

[11] Kawel‐Boehm N , Hetzel SJ , Ambale‐Venkatesh B , Captur G , Francois CJ , Jerosch‐Herold M , Salerno M , Teague SD , Valsangiacomo‐Buechel E , Geest Rob J , et al. Reference ranges (“normal values”) for cardiovascular magnetic resonance (CMR) in adults and children: 2020 update. J Cardiovasc Magn Reson. 2020;22:1–63. doi: 10.1186/s12968-020-00683-3 33308262 PMC7734766

[12] Pirruccello JP , Di Achille P , Nauffal V , Nekoui M , Friedman SF , Klarqvist MDR , Chaffin MD , Weng LC , Cunningham JW , Khurshid S , et al. Genetic analysis of right heart structure and function in 40,000 people. Nat Genet. 2022;54:792–803. doi: 10.1038/s41588-022-01090-3 35697867 PMC10313645

[13] Aung N , Vargas JD , Yang C , Fung K , Sanghvi MM , Piechnik SK , Neubauer S , Manichaikul A , Rotter JI , Taylor KD , et al. Genome‐wide association analysis reveals in‐ sights into the genetic architecture of right ventricular structure and function. Nat Genet. 2022;54:783–791. doi: 10.1038/s41588-022-01083-2 35697868 PMC11929962

[14] Ahlberg G , Andreasen L , Ghouse J , Bertelsen L , Bundgaard H , Haunsø S , Svendsen JH , Olesen MS . Genome‐wide association study identifies 18 novel loci associated with left atrial volume and function. Eur Heart J. 2021;42:4523–4534. doi: 10.1093/eurheartj/ehab466 34338756 PMC8633773

[15] Aung N , Vargas JD , Yang C , Cabrera CP , Warren HR , Fung K , Tzanis E , Barnes MR , Rotter JI , Taylor KD , et al. Genome‐wide analysis of left ventricular image‐ derived phenotypes identifies fourteen loci associated with cardiac morphogenesis and heart failure development. Circulation. 2019;140:1318–1330. doi: 10.1161/CIRCULATIONAHA.119.041161 31554410 PMC6791514

[16] Aung N , Wang Q , Duijvenboden S , Burns R , Stoma S , Raisi‐Estabragh Z , Ahmet S , Allara E , Wood A , Di Angelantonio E , et al. Association of longer leukocyte telomere length with cardiac size, function, and heart failure. JAMA Cardiol. 2023;8:808–815. doi: 10.1001/jamacardio.2023.2167 37494011 PMC10372756

[17] Burgess S , Davies NM , Dudbridge F , Gill D , Glymour MM , Hartwig FP , Kutalik Z , Holmes MV , Minelli C , Morrison JV , et al. Guidelines for performing Mendelian randomization investigations: update for summer 2023. Wellcome Open Research. 2023;4:186. doi: 10.12688/wellcomeopenres.15555.3 32760811 PMC7384151

[18] Chen SY , Feng Z , Yi X . A general introduction to adjustment for multiple comparisons. J Thorac Dis. 2017;9:1725–1729. doi: 10.21037/jtd.2017.05.34 28740688 PMC5506159

[19] Francis CM , Futschik ME , Huang J , Bai W , Sargurupremraj M , Teumer A , Breteler MMB , Petretto E , Ho ASR , Amouyel P , et al. Genome‐wide associations of aortic distensibility suggest causality for aortic aneurysms and brain white matter hyperintensities. Nat Commun. 2022;13:4505. doi: 10.1038/s41467-022-32219-x 35922433 PMC9349177

[20] Fung K , Ramírez J , Warren HR , Aung N , Lee AM , Tzanis E , Petersen SE , Munroe PB . Genome‐wide association study identifies loci for arterial stiffness index in 127,121 UK Biobank participants. Sci Rep. 2019;9:9143. doi: 10.1038/s41598-019-45703-0 31235810 PMC6591384

[21] Li C , Stoma S , Lotta LA , Warner S , Albrecht E , Allione A , Arp PP , Broer L , Buxton JL , Alves ADSC , et al. Genome‐wide association analysis in humans links nucleotide metabolism to leukocyte telomere length. Am J Hum Genet. 2020;106:389–404. doi: 10.1016/j.ajhg.2020.02.006 32109421 PMC7058826

[22] Codd V , Nelson CP , Albrecht E , Mangino M , Deelen J , Buxton JL , Hottenga JJ , Fischer K , Esko T , Surakka I , et al. Identification of seven loci affecting mean telomere length and their association with disease. Nat Genet. 2013;45:422–427. doi: 10.1038/ng.2528 23535734 PMC4006270

[23] Mangino M , Hwang SJ , Spector TD , Hunt SC , Kimura M , Fitzpatrick AL , Christiansen L , Petersen I , Elbers CC , Harris T , et al. Genome‐wide meta‐analysis points to CTC1 and ZNF676 as genes regulating telomere homeostasis in humans. Hum Mol Genet. 2012;21:5385–5394. doi: 10.1093/hmg/dds382 23001564 PMC3510758

[24] Kuo CL , Pilling LC , Kuchel George A , Luigi F , David M . Telomere length and aging‐ related outcomes in humans: a Mendelian randomization study in 261,000 older participants. Aging Cell. 2019;18:e13017. doi: 10.1111/acel.13017 31444995 PMC6826144

[25] Davies NM , Holmes MV , Smith GD . Reading Mendelian randomisation studies: a guide, glossary, and checklist for clinicians. BMJ. 2018;362:k601. doi: 10.1136/bmj.k601 30002074 PMC6041728

[26] Salih A , Galazzo IB , Petersen SE , Lekadir K , Radeva P , Menegaz G , Altmann A . Telomere length is causally connected to brain MRI image derived phenotypes: a Mendelian randomization study. PLoS One. 2022;17:e0277344. doi: 10.1371/journal.pone.0277344 36399449 PMC9674175

[27] Yates AD , Achuthan P , Akanni W , Allen J , Allen J , Alvarez‐Jarreta J , Amode MR , Armean IM , Azov AG , Bennett R , et al. Ensembl 2020. Nucleic Acids Res. 2020;48:D682–D688. doi: 10.1093/nar/gkz966 31691826 PMC7145704

[28] Pooley KA , Bojesen SE , Weischer M , Nielsen SF , Thompson D , Amin Al Olama A , Michailidou K , Tyrer JP , Benlloch S , Brown J , et al. A genome‐wide association scan (GWAS) for mean telomere length within the COGS project‐: identified loci show little association with hormone‐related cancer risk. Hum Mol Genet. 2013;22:5056–5064. doi: 10.1093/hmg/ddt355 23900074 PMC3836481

[29] Hemani G , Zheng J , Elsworth B , Wade KH , Haberland V , Baird D , Laurin C , Burgess S , Bowden J , Langdon R , et al. The MR‐Base platform supports systematic causal inference across the human phenome. Elife. 2018;7:e34408. doi: 10.7554/eLife.34408 29846171 PMC5976434

[30] Bowden J , Davey SG , Haycock PC , Burgess S . Consistent estimation in Mendelian randomization with some invalid instruments using a weighted median estimator. Genet Epidemiol. 2016;40:304–314. doi: 10.1002/gepi.21965 27061298 PMC4849733

[31] Hartwig FP , Davey SG , Bowden J . Robust inference in summary data Mendelian randomization via the zero modal pleiotropy assumption. Int J Epidemiol. 2017;46:1985–1998. doi: 10.1093/ije/dyx102 29040600 PMC5837715

[32] Bowden J , Davey SG , Burgess S . Mendelian randomization with invalid instruments: effect estimation and bias detection through Egger regression. Int J Epidemiol. 2015;44:512–525. doi: 10.1093/ije/dyv080 26050253 PMC4469799

[33] Verbanck M , Chen CY , Neale B , Do R . Detection of widespread horizontal pleiotropy in causal relationships inferred from Mendelian randomization between complex traits and diseases. Nat Genet. 2018;50:693–698. doi: 10.1038/s41588-018-0099-7 29686387 PMC6083837

[34] Benjamini Y , Hochberg Y . Controlling the false discovery rate: a practical and powerful approach to multiple testing. J R Statist Soc B. 1995;57:289–300. doi: 10.1111/j.2517-6161.1995.tb02031.x

[35] Locke AE , Kahali B , Berndt SI , Justice AE , Pers TH , Day FR , Powell C , Vedantam S , Buchkovich ML , Yang J , et al. Genetic studies of body mass index yield new insights for obesity biology. Nature. 2015;518:197–206. doi: 10.1038/nature14177 25673413 PMC4382211

[36] Liu M , Jiang Y , Wedow R , Li Y , Brazel DM , Chen F , Datta G , Davila‐Velderrain J , McGuire D , Tian C , et al. Association studies of up to 1.2 million individuals yield new insights into the genetic etiology of tobacco and alcohol use. Nat Genet. 2019;51:237–244. doi: 10.1038/s41588-018-0307-5 30643251 PMC6358542

[37] Cheng S , Fernandes VR , Bluemke DA , McClelland RL , Kronmal RA , Lima JAC . Age‐related left ventricular remodeling and associated risk for cardiovascular outcomes: the Multi‐Ethnic Study of Atherosclerosis. Circ Cardiovasc Imaging. 2009;2:191–198. doi: 10.1161/CIRCIMAGING.108.819938 19808592 PMC2744970

[38] Anversa P , Hiler B , Ricci R , Guideri G , Olivetti G . Myocyte cell loss and my ocyte hypertrophy in the aging rat heart. J Am Coll Cardiol. 1986;8:1441–1448. doi: 10.1016/S0735-1097(86)80321-7 2946746

[39] Nethononda RM , Lewandowski AJ , Stewart R , Kylinterias I , Whitworth P , Francis J , Leeson P , Watkins H , Neubauer S , Rider OJ . Gender‐specific patterns of age‐related decline in aortic stiffness: a cardiovascular magnetic resonance study including normal ranges. J Cardiovasc Magn Reson. 2015;17:20. doi: 10.1186/s12968-015-0126-0 25827408 PMC4332729

[40] Kass DA . Age‐related changes in venticular–arterial coupling: pathophysiologic implications. Heart Fail Rev. 2002;7:51–62. doi: 10.1023/A:1013749806227 11790922

[41] Hiam D , Smith C , Voisin S , Denham J , Yan X , Landen S , Jacques M , Alvarez‐Romero J , Garnham A , Woessner MN , et al. Aerobic capacity and telomere length in human skeletal muscle and leukocytes across the lifespan. Aging (Albany NY). 2020;12:359–369. doi: 10.18632/aging.102627 31901896 PMC6977669

[42] Vaiserman A , Krasnienkov D . Telomere length as a marker of biological age: state‐of‐the‐art, open issues, and future perspectives. Front Genet. 2021;11:630186. doi: 10.3389/fgene.2020.630186 33552142 PMC7859450

[43] Cheng F , Luk AO , Shi M , Huang C , Jiang G , Yang A , Wu H , Lim CKP , Tam CHT , Fan B , et al. Shortened leukocyte telomere length is associated with glycemic progression in type 2 diabetes: a prospective and mendelian randomization analysis. Diabetes Care. 2022;45:701–709. doi: 10.2337/dc21-1609 35085380 PMC8918237

[44] Grunnet LG , Pilgaard K , Alibegovic A , Jensen CB , Hjort L , Ozanne SE , Martin B , Vaag A , Brøns C . Leukocyte telomere length is associated with elevated plasma glucose and HbA1c in young healthy men independent of birth weight. Sci Rep. 2019;9:7639. doi: 10.1038/s41598-019-43387-0 31113969 PMC6529491

[45] Martens DS , Sleurs H , Dockx Y , Rasking L , Plusquin M , Nawrot TS . Association of newborn telomere length with blood pressure in childhood. JAMA Netw Open. 2022;5:e2225521. doi: 10.1001/jamanetworkopen.2022.25521 35930283 PMC9356312

[46] Aviv A , Aviv H . Telomeres and essential hypertension. Am J Hypertens. 1999;12:427–432. doi: 10.1016/S0895-7061(98)00202-7 10232505

[47] Karimi B , Yunesian M , Nabizadeh R , Mehdipour P . Serum level of total lipids and telomere length in the male population: a cross‐sectional study. Am J Mens Health. 2019;13:1557988319842973. doi: 10.1177/1557988319842973 30961458 PMC6457029

[48] Qin X , Cong Z , Fei B . Automatic segmentation of right ventricular ultrasound images using sparse matrix transform and a level set. Phys Med Biol. 2013;58:7609–7624. doi: 10.1088/0031-9155/58/21/7609 24107618 PMC3925785

[49] Ding H , Yan F , Zhou LL , Ji XH , Gu XN , Tang ZW , Chen RH . Association between previously identified loci affecting telomere length and coronary heart disease (CHD) in Han Chinese population. Clin Interv Aging. 2014;9:857. doi: 10.2147/CIA.S60760 24904205 PMC4041376

[50] Song Y , Yan M , Li J , Li J , Jin T , Chen C . Association between TNIP1, MPHOSPH6 and ZNF208 genetic polymorphisms and the coronary artery disease risk in Chinese Han population. Oncotarget. 2017;8:77233–77240. doi: 10.18632/oncotarget.20432 29100383 PMC5652776

[51] Willer CJ , Schmidt E , Sengupta S , Peloso GM , Gustafsson S , Kanoni S , Ganna A , Chen J , Buchkovich ML , Mora S , et al. Discovery and refinement of loci associated with lipid levels. Nat Genet. 2013;45:1274–1283. 24097068 10.1038/ng.2797 PMC3838666

[52] Lu S , Zhong J , Wu M , Huang K , Zhou Y , Zhong Z , Li Q , Zhou H . Genetic analysis of the relation of telomere length‐related gene (RTEL1) and coronary heart disease risk. Mol Genet Genomic Med. 2019;7:e550. doi: 10.1002/mgg3.550 30623606 PMC6418357

