# Supplementary material for: Leukocyte Telomere Length and Cardiac Structure and Function: A Mendelian Randomization Study
Source: J Am Heart Assoc. 2024 Jan 31;13(3):e032708. doi: 10.1161/JAHA.123.032708 (PMC11056120; doi:10.1161/JAHA.123.032708)
Supplement: Supplementary file 1 — Tables S1–S2 [file JAH3-13-e032708-s001.pdf]

SUPPLEMENTAL MATERIAL

Table S1. **The results of the two sample MR in the primary analysis.** RVESV: right ventricular end systolic volume; RVSV: right ventricular stroke volume; RVEF: right ventricular ejection fraction; RVEDV: right ventricular end diastolic volume; LVEDV: left ventricular end-diastolic volume; LVM: left ventricular mass; LVESV: left ventricular end-systolic volume; LVEF: left ventricular ejection fraction; LVMVR: left ventricular mass to end-diastolic volume ratio; LVSV: left ventricular stroke volume; PA: pulmonary artery.

CMR Metrics	Beta (IVW)	P-value	Study	Sample size
Ascending aortic distensibility	-0.0539844	0.079	Francis, C. M., Futschik, M. E., Huang, J., Bai, W., Sargurupremraj, M., Teumer, A., ... & Matthews, P. M. (2022). Genome-wide associations of aortic distensibility suggest causality for aortic aneurysms and brain white matter hyperintensities. Nature communications, 13(1), 4505.	32,590
Ascending aortic maximum area	11.54794	0.256		
Ascending aortic minimum area	10.98852	0.25		
Descending aortic distensibility	-0.032088	0.3		
Descending aortic maximum area	1.4957458	0.649		
Descending aortic minimum area	1.293896	0.6716		
Arterial stiffness index	0.0308142	0.234	Fung, Kenneth, et al. "Genome-wide association study identifies loci for arterial stiffness index in 127,121 UK Biobank participants." Scientific reports 9.1 (2019): 9143.	127,121
Indexed left atrial maximum volume	0.0418936	0.336	Ahlberg, Gustav, et al. "Genome-wide association study identifies 18 novel loci associated with left atrial volume and function." European Heart Journal 42.44 (2021): 4523-4534.	35,658
Indexed left atrial minimum volume	0.0446609	0.343		
Left atrial active emptying fraction	-0.0336316	0.38		
Left atrial passive emptying fraction	0.045182	0.808		
Left atrial total emptying fraction	-0.029284	0.486		
Left ventricular end-diastolic volume	0.1379894	0.011	Aung, Nay, et al. "Genome-wide analysis of left ventricular image-derived phenotypes identifies fourteen loci associated with cardiac morphogenesis and heart failure development." Circulation 140.16 (2019): 1318-1330.	16,923
Left ventricular ejection fraction	-0.0410226	0.453		
Left ventricular end-systolic volume	0.1181972	0.029		
Left ventricular mass to end-diastolic volume ratio	0.0595421	0.368		
Left ventricular mass	0.2166963	0.0003		
invnorm_lvef	-0.0069353	0.833	Pirruccello, James P., et al. "Genetic analysis of right heart structure and function in 40,000 people." Nature genetics 54.6 (2022): 792-803.	40,000
invnorm_lv_sv_indexed	0.0562995	0.183		
invnorm_max_aa_diam	0.0833539	0.123		
invnorm_max_aa_diam_indexed	0.0959076	0.054		

invnorm_max_lv	0.0315652	0.402		
invnorm_max_lv_indexed	0.0617218	0.157		
invnorm_min_lv	0.0233792	0.454		
invnorm_min_lv_indexed	0.0446598	0.211		
invnorm_pa_ao	0.0186648	0.716		
invnorm_pa_ao_diastole	-0.0253027	0.621		
invnorm_rafac	0.0551622	0.221		
invnorm_ramax_area	0.0133085	0.705		
invnorm_ramax_area_indexed	0.0359005	0.33		
invnorm_ramin_area	-0.0171331	0.637		
invnorm_ramin_area_indexed	-0.0036021	0.919		
invnorm_RVEDV	0.0238742	0.509		
invnorm_RVEDV_indexed	0.0446099	0.204		
invnorm_RVEDV_LVEDV_ratio	-0.0146684	0.709		
invnorm_RVEF	-0.0005869	0.986		
invnorm_RVEF_LVEF_ratio	0.0129976	0.716		
invnorm_RVESV	0.014848	0.621		
invnorm_RVESV_indexed	0.0344328	0.332		
invnorm_RVESV_LVESV_ratio	-0.0251037	0.481		
invnorm_RVSV	0.0233202	0.566		
invnorm_RVSV_indexed	0.0560061	0.246		
invnorm_RVSV_LVSV_ratio	-0.0105078	0.766		
invnorm_short_axis_cm_pa	0.0854474	0.049		
invnorm_short_axis_cm_pa_diastole	0.0336544	0.521		
invnorm_short_axis_cm_pa_diastole_indexed	0.0502536	0.321		
invnorm_short_axis_cm_pa_indexed	0.1033347	0.007		
invnorm_short_axis_cm_pa_strain	0.0275412	0.502		
invnorm_short_axis_cm_root	0.0305874	0.33		
invnorm_short_axis_cm_root_indexed	0.0570327	0.166		

RVEDV	0.0666283	0.139	Aung, Nay, et al. "Genome-wide association analysis reveals insights into the genetic architecture of right ventricular structure and function." Nature genetics 54.6 (2022): 783-791.	29,506
RVEF	0.004781	0.907		
RVESV	0.0451469	0.326		
RVSV	0.062173	0.134		

Table S2. **Significant associations based on screening each instrumental variable against the GWAS summary statistics in GWAS Catalog.**

SNP	P-value	Mapped genes	Trait name	Accession Id	IV_ID
rs2695242	-	-	-	-	1
rs11125529	9.00E-16	ACYP2	Telomere length	GCST90103979	2
rs11125529	8.00E-10	ACYP2	Telomere length	GCST001936	2
rs6772228	-	-	-	-	3
rs55749605	2.00E-08	FAM172BP,SENP7	Leukocyte telomere length	GCST009856	4
rs55749605	4.00E-08	FAM172BP,SENP7	Asthma	GCST010043	4
rs7643115	-	-	-	-	5
rs13137667	2.00E-08	MOB1B	Leukocyte telomere length	GCST009856	6
rs7675998	5.00E-22	MIR4454,TOMM22P4	Telomere length	GCST90103979	7
rs7705526	6.00E-163	TERT	Mean corpuscular volume	GCST90002338	8
rs7705526	8.00E-137	TERT	Mean corpuscular hemoglobin	GCST90002326	8
rs7705526	3.00E-122	TERT	Mean corpuscular volume	GCST90002334	8
rs7705526	5.00E-108	TERT	Red blood cell count	GCST90002367	8
rs7705526	3.00E-105	TERT	Mean corpuscular volume	GCST90018966	8
rs7705526	2.00E-103	TERT	Mean corpuscular hemoglobin	GCST90002322	8
rs7705526	1.00E-98	TERT	Mean corpuscular hemoglobin	GCST007068	8
rs7705526	2.00E-95	TERT	Mean corpuscular hemoglobin	GCST90018964	8
rs7705526	2.00E-92	TERT	Telomere length	GCST90103979	8
rs7705526	1.00E-90	TERT	Platelet count	GCST90018969	8
rs7705526	5.00E-88	TERT	Platelet count	GCST90002357	8
rs7705526	3.00E-81	TERT	Red blood cell count	GCST90002363	8
rs7705526	2.00E-69	TERT	Red blood cell count	GCST007069	8

rs7705526	4.00E-67	TERT	Leukocyte telomere length	GCST008366	8
rs7705526	4.00E-65	TERT	Lung adenocarcinoma	GCST012201	8
rs7705526	4.00E-61	TERT	Neutrophil count	GCST90002355	8
rs7705526	2.00E-58	TERT	Plateletcrit	GCST90002400	8
rs7705526	2.00E-58	TERT	Mean corpuscular volume	GCST90018746	8
rs7705526	2.00E-57	TERT	Mean corpuscular volume	GCST90056174	8
rs7705526	5.00E-54	TERT	Myeloproliferative neoplasms	GCST90000032	8
rs7705526	9.00E-54	TERT	Neutrophil count	GCST90002351	8
rs7705526	2.00E-52	TERT	White blood cell count	GCST90002378	8
rs7705526	3.00E-52	TERT	Lung adenocarcinoma	GCST008836	8
rs7705526	7.00E-49	TERT	Mean corpuscular hemoglobin	GCST90018744	8
rs7705526	4.00E-45	TERT	Neutrophil count	GCST90018968	8
rs7705526	5.00E-45	TERT	Leukocyte telomere length	GCST009856	8
rs7705526	5.00E-45	TERT	Mean corpuscular volume	GCST90002392	8
rs7705526	8.00E-43	TERT	White blood cell count	GCST90002374	8
rs7705526	1.00E-42	TERT	Non-small cell lung cancer	GCST012199	8
rs7705526	2.00E-41	TERT	Plateletcrit	GCST004607	8
rs7705526	2.00E-41	TERT	Platelet count	GCST90018749	8
rs7705526	4.00E-41	TERT	Non-small cell lung cancer	GCST008834	8
rs7705526	2.00E-39	TERT	Mean corpuscular hemoglobin	GCST90002323	8
rs7705526	2.00E-38	TERT	Mean corpuscular hemoglobin	GCST90002390	8
rs7705526	3.00E-38	TERT	Leukocyte telomere length	GCST008366	8
rs7705526	8.00E-37	TERT	Mean spheric corpuscular volume	GCST90002397	8
rs7705526	4.00E-35	TERT	Lung adenocarcinoma	GCST004744	8
rs7705526	4.00E-34	TERT	Platelet count	GCST90002402	8
rs7705526	1.00E-32	TERT	Red blood cell count	GCST90002403	8
rs7705526	1.00E-32	TERT	Platelet count	GCST90056183	8
rs7705526	7.00E-32	TERT	Mean reticulocyte volume	GCST90002396	8
rs7705526	8.00E-32	TERT	Leukocyte telomere length	GCST90278198	8
rs7705526	4.00E-29	TERT	White blood cell count	GCST007070	8

rs7705526	4.00E-22	TERT	Neutrophil count	GCST90002398	8
rs7705526	2.00E-20	TERT	Low-grade serous and serous borderline ovarian cancer	GCST004481	8
rs7705526	8.00E-20	TERT	Neutrophil count	GCST004629	8
rs7705526	1.00E-19	TERT	Sum basophil neutrophil counts	GCST004620	8
rs7705526	6.00E-19	TERT	Myeloid white cell count	GCST004626	8
rs7705526	6.00E-19	TERT	Serous borderline ovarian cancer	GCST004479	8
rs7705526	6.00E-19	TERT	Sum neutrophil eosinophil counts	GCST004613	8
rs7705526	1.00E-18	TERT	Granulocyte count	GCST004614	8
rs7705526	8.00E-18	TERT	White blood cell count	GCST90002407	8
rs7705526	8.00E-18	TERT	White blood cell count	GCST004610	8
rs7705526	6.00E-15	TERT	Mitochondrial DNA copy number	GCST90026371	8
rs7705526	2.00E-14	TERT	Cutaneous malignant melanoma	GCST010304	8
rs7705526	1.00E-12	TERT	Neutrophil count	GCST90056178	8
rs7705526	2.00E-12	TERT	Basal cell carcinoma	GCST90013410	8
rs7705526	5.00E-12	TERT	Serum levels of protein THPO	GCST90089245	8
rs7705526	1.00E-11	TERT	Neutrophil percentage of white cells	GCST90002399	8
rs7705526	6.00E-10	TERT	Serous invasive ovarian cancer	GCST004478	8
rs7705526	6.00E-10	TERT	Chronic lymphocytic leukemia	GCST004146	8
rs7705526	7.00E-10	TERT	Invasive epithelial ovarian cancer	GCST004415	8
rs7705526	8.00E-10	TERT	Eosinophil percentage of white cells	GCST90002382	8
rs7705526	1.00E-09	TERT	Hypothyroidism	GCST007073	8
rs7705526	2.00E-09	TERT	Peripheral blood CD34+ cell levels	GCST90102460	8
rs7705526	3.00E-09	TERT	Mitochondrial DNA copy number (adjusted)	GCST90268497	8
rs7705526	4.00E-09	TERT	DNA methylation Hannum age acceleration	GCST90014301	8
rs7705526	2.00E-08	TERT	High-grade serous ovarian cancer	GCST004480	8
rs34991172	6.00E-09	CARMIL1	Leukocyte telomere length	GCST009856	9
rs805297	3.00E-10	-	Rheumatoid arthritis	GCST001203	10
rs59294613	6.00E-25	POT1	Telomere length	GCST90103979	11
rs59294613	1.00E-13	POT1	Leukocyte telomere length	GCST009856	11

rs9419958	9.00E-83	STN1	Telomere length	GCST90103979	12
rs9419958	5.00E-19	STN1	Leukocyte telomere length	GCST009856	12
rs9419958	1.00E-16	STN1	Uterine fibroids	GCST009158	12
rs9419958	9.00E-11	STN1	Telomere length	GCST001697	12
rs9419958	5.00E-10	STN1	Basal cell carcinoma	GCST008871	12
rs228595	2.00E-13	ATM	Telomere length	GCST90103979	13
rs228595	1.00E-08	ATM	Leukocyte telomere length	GCST009856	13
rs76891117	1.00E-10	DCAF4	Systolic blood pressure	GCST007087	14
rs3785074	5.00E-14	TERF2	Telomere length	GCST90103979	15
rs3785074	5.00E-10	TERF2	Leukocyte telomere length	GCST009856	15
rs62053580	4.00E-11	RFWD3	Telomere length	GCST90103979	16
rs62053580	4.00E-08	RFWD3	Leukocyte telomere length	GCST009856	16
rs7194734	2.00E-23	MPHOSPH6	Height	GCST90018959	17
rs7194734	3.00E-13	MPHOSPH6	Telomere length	GCST90103979	17
rs7194734	7.00E-10	MPHOSPH6	Leukocyte telomere length	GCST009856	17
rs3027234	2.00E-08	CTC1	Telomere length	GCST001697	18
rs8105767	4.00E-18	ZNF257,ZNF208	Telomere length	GCST90103979	19
rs8105767	5.00E-13	ZNF257,ZNF208	Leukocyte telomere length	GCST009856	19
rs8105767	1.00E-08	ZNF257,ZNF208	Uterine leiomyomata	GCST90239856	19
rs8105767	5.00E-08	ZNF257,ZNF208	Uterine fibroids	GCST90018934	19
rs6028466	3.00E-07	HSPE1P1,RN7SL680P	Telomere length	GCST001024	20
rs71325459	5.00E-08	STMN3,MHENCR	Age at menopause	GCST007079	21
rs75691080	7.00E-17	MHENCR,STMN3	Telomere length	GCST90103979	22
rs75691080	6.00E-14	MHENCR,STMN3	Leukocyte telomere length	GCST009856	22
rs75691080	2.00E-08	MHENCR,STMN3	Uterine leiomyomata	GCST90239856	22
rs73624724	6.00E-12	ZBTB46	Leukocyte telomere length	GCST009856	23
